# Antiviral Effects of Polyphenols from Marine Algae

**DOI:** 10.3390/biomedicines9020200

**Published:** 2021-02-17

**Authors:** Natalya N. Besednova, Boris G. Andryukov, Tatyana S. Zaporozhets, Sergey P. Kryzhanovsky, Ludmila N. Fedyanina, Tatyana A. Kuznetsova, Tatyana N. Zvyagintseva, Mikhail Yu. Shchelkanov

**Affiliations:** 1 G.P. Somov Institute of Epidemiology and Microbiology, Russian Federal Service for Surveillance on Consumer Rights Protection and Human Wellbeing, 690087 Vladivostok, Russia; andrukov_bg@mail.ru (B.G.A.); niiem_vl@mail.ru (T.S.Z.); takuznets@mail.ru (T.A.K.); adorob@mail.ru (M.Y.S.); 2 School of Biomedicine, Far Eastern Federal University (FEFU), 690091 Vladivostok, Russia; fedyanina.ln@dvfu.ru; 3 Medical Association of the Far Eastern Branch of the Russian Academy of Sciences, 690022 Vladivostok, Russia; priemmodvoran@mail.ru; 4 Elyakov Pacific Institute of Bioorganic Chemistry, FEB RAS, 690022 Vladivostok, Russia; zvyag@piboc.dvo.ru; 5 Federal Scientific Center of the Eastern Asia Terrestrial Biodiversity, Far Eastern Branch of Russian Academy of Sciences, 690091 Vladivostok, Russia; 6 National Scientific Center of Marine Biology, Far Eastern Branch of Russian Academy of Sciences, 690091 Vladivostok, Russia

**Keywords:** polyphenols, flavonoids, antioxidants, marine algae, anti-viral activity, mechanism of action

## Abstract

The disease-preventive and medicinal properties of plant polyphenolic compounds have long been known. As active ingredients, they are used to prevent and treat many noncommunicable diseases. In recent decades, marine macroalgae have attracted the attention of biotechnologists and pharmacologists as a promising and almost inexhaustible source of polyphenols. This heterogeneous group of compounds contains many biopolymers with unique structure and biological properties that exhibit high anti-infective activity. In the present review, the authors focus on the antiviral potential of polyphenolic compounds (phlorotannins) from marine algae and consider the mechanisms of their action as well as other biological properties of these compounds that have effects on the progress and outcome of viral infections. Effective nutraceuticals, to be potentially developed on the basis of algal polyphenols, can also be used in the complex therapy of viral diseases. It is necessary to extend in vivo studies on laboratory animals, which subsequently will allow proceeding to clinical tests. Polyphenolic compounds have a great potential as active ingredients to be used for the creation of new antiviral pharmaceutical substances.

## 1. Introduction

The high virulence of new and recurring viruses and the lack of effective treatments for the diseases caused by them pose a serious challenge to public health systems. The development of highly effective broad-spectrum antiviral drugs with low toxicity and low cost has been one of the major issues in virology and pharmaceutics for many years. In the period of the ongoing COVID-19 pandemic, it has acquired particular relevance and importance and is aimed at creating agents that inhibit the entry and replication of the virus while modulating the body’s defence systems.

The virus reproduction process includes three phases [1]. The first one is adsorption and entry of the virus into the cell, the release of its internal structural components, and modification into a state in which it can cause an infectious process. The attachment of the virus to macroorganism host cells is a specific interaction between the surface proteins of the virus and the receptors located on the surface of host cells. The second phase of reproduction is regulated by complex processes with the expression of the viral genome. Finally, the third stage of reproduction is the release of viral offspring out of the host cell by budding or lysis.

Currently, medicine has a large range of antiviral agents that can have an effect on each of these stages [2]. At the same time, a rapid increase in their number is observed annually due to compounds isolated from terrestrial plants. The possibility of using synthetic and herbal preparations for the treatment of viral diseases is determined by a number of properties, such as a therapeutic effect, the absence or minimum of side reactions, and low toxicity.

Synthetic antiviral drugs act faster and provide, as a rule, the maximum therapeutic effect. However, their disadvantage is a large number of contraindications and side reactions, as well as addiction and the absence of the desired effect in the future. Herbal antiviral drugs have a wide spectrum of action (apart from the antiviral effect, they have anti-inflammatory, antioxidant and immunomodulatory effects), are less toxic or non-toxic in working doses, and have minimal side effects. It is possible that herbal medicine may have potential as a prophylactic agent and even a therapeutic agent for patients with viral infection.

Despite certain advances in chemotherapy of viral diseases, clinical practice faces serious problems such as the emergence of drug-resistant variants of viruses and side effects of antiviral medicines. This circumstance dictates the need to develop new antiviral drugs with different mechanisms of action [3,4].

Studies on compounds with antiviral properties derived from terrestrial and marine plants have shown that, due to their diverse mechanisms of action (antiviral, immunostimulatory, anti-inflammatory and antioxidant), viruses, as a rule, do not acquire resistance to these compounds. Therefore, aquatic organisms producing substances that are sometimes not found in terrestrial plants and have extremely high polyvalent biological activity have attracted the special attention of researchers [5].

The world’s experience in using marine-derived pharmaceuticals shows the enormous potential of marine organisms as raw materials for the creation of original pharmaceutical substances and medicines [6]. Algae, sponges, bacteria, fungi, invertebrates, soft corals, fish, etc. can be sources of new antiviral pharmacological compounds of marine origin [7,8,9]. A number of compounds from these organisms are commercially available on the pharmaceutical market worldwide as an alternative to antiviral drugs [10].

The purpose of this review is to summarise the literature data on the antiviral potential of polyphenolic compounds of seaweed, to highlight the mechanisms of their action and to characterise the other biological properties of these compounds that affect the course and outcome of viral infections. The authors draw the attention of researchers to the fact that algae are an extremely promising source of antiviral compounds, and research in this direction should be continued.

## 2. General Characteristics of the Polyphenolic Compounds of Seaweed

Marine macroalgae are a unique raw material for obtaining a wide range of natural compounds with interesting and useful biological properties. Their composition is characterised by a rich content of mineral and organic substances. For thousands of years, these hydrobionts have been actively used by humans and animals for food and have served as a valuable source of proteins, fats, carbohydrates, dietary fibre, minerals, etc.

Regular consumption of seaweed can reduce the risk of various pathologies, including cancer, metabolic and degenerative disorders, infectious diseases and cardiovascular diseases. The highest antiviral activity, as shown by numerous experimental studies, is possessed by polyphenolic compounds and sulphated polysaccharides. The content of biologically active substances in seaweed varies depending on the season and region of collection and is largely determined by the type of algae. According to the presence of specific pigments, macroalgae are divided into three main groups: brown (Phaeophyceae), green (Chlorophyta) and red (Rhodophyta) seaweed.

**Figure 1:**
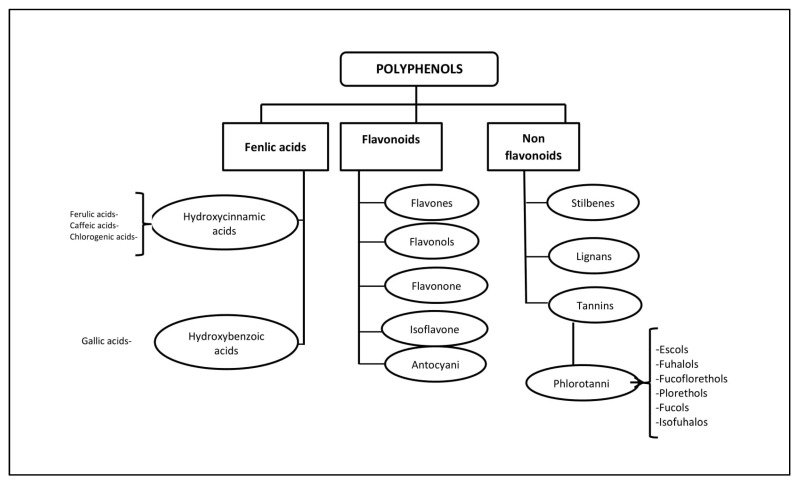
Classification of polyphenols and six main subclasses of seaweed phlorotannins.

Polyphenols (PPs)—highly hydrophilic secondary metabolites of seaweed—are one of the most numerous groups of substances in the plant kingdom. Macro- and microalgae, as well as cyanobacteria accumulate PPs, in particular, phloroglucinol and its polymers, i.e., phlorotannins [11]. Bromophenols, phenolic acids and flavonoids account for the largest proportion of phenolic compounds found in red and green seaweed [12]. Phlorotannins (PTs) are a heterogeneous group of unique polyphenolic compounds differing in structure and degree of polymerisation and are found only in brown seaweed (up to 25% of dry weight) [13,14]. The largest amount of PTs accumulates in fucus brown seaweed [15,16,17,18]. PTs consist of monomeric units of phloroglucinol (1,3,5-hydroxybenzene), from which more than 700 natural variations of these compounds have been obtained and used in various fields [19] (Figure 1).

Unlike the tannins of terrestrial plants, PTs have a wider range of molecular weights, from 126 Da to 650 kDa (more often from 10 to 100 kDa). The characterisation of PPs is difficult due to heterogeneity both in molecular weight and in the level of isomerisation [20,21]. There is still little information about endogenous digestion and microbial catabolism of these compounds [22]. It is known that about 90–95% of dietary PPs reach the intestine unchanged [23], where, as a result of metabolism and biotransformation, low molecular weight compounds with less chemical heterogeneity are formed than in the original [24].

Some PTs in seaweed can be sulphated or halogenated [25]. The biosynthesis of PTs is carried out through the acetate-malonate pathway in the Golgi apparatus in the perinuclear region of the cell. They are usually not secreted, and cell destruction is necessary to obtain them. In terms of structure and polymeric properties, PTs represent an extensive group of molecules that differ in the nature of the bonds between phloroglucinol and hydroxyl groups (Figure 1). Depending on the type of bond between the monomers, phlorotannins are divided into four subclasses: phlorethols and fuhalols, fucols, fucophlorethols, and eckols and carmalol [26,27]. These compounds exist mainly in a soluble form or in a bound state with components of the cell wall, which ensure its integrity as well as protection from herbivores and oxidative stress.

Terrestrial plants produce tannins that are composed of only three or four phenolic rings, while seaweed PTs are composed of eight phenolic rings. PTs have very strong antioxidant properties as phenolic rings act as electron traps for free radicals [12]. A positive correlation has been noted between the antioxidant activity of PTs and the number of hydroxyl groups present in the structure of the compound [28]. PTs inhibit α-glucosidase, which is responsible for the stepwise removal of terminal glucose residues from the N-glycan chains associated with glycoprotein maturation. Most glycoproteins of the viral environment contain N-linked glycans, and α-glucosidase inhibitors have been proposed as useful broad-spectrum antiviral agents based on their activity against enveloped viruses [29]. The anti-inflammatory [30], antiallergic [31], antiviral [32] and antitumor [33] properties, as well as antidiabetic and radioprotective effects [34] of these biologically active compounds have been demonstrated.

Methods for obtaining PTs, their identification and establishment of the structure are described in sufficient detail in numerous works [13,14,35]. The main difficulty in the extraction of PPs arises from their presence in the form of complex polymer mixtures, for example, with polysaccharides, which, along with proteins, are the main covalently bound component of the algal cell wall [36].

## 3. Interaction of Seaweed Polyphenols with Enveloped and Nonenveloped Viruses

Resistance of viruses to adverse environmental factors is determined by their structure. There are viruses with simple and complex structure. Simple, or nonenveloped, viruses are composed of a nucleic acid and protein envelope (capsid). Complex, or enveloped, viruses are surrounded by a lipoprotein envelope (supercapsid) over the capsid, which makes them more vulnerable to adverse environmental factors [5,6,23].

Enveloped and nonenveloped viruses also differ in resistance to chemicals, including disinfectants. Thus, the lipoprotein-enveloped influenza, parainfluenza viruses and coronaviruses are low-resistant pathogens; adenoviruses are more resistant; and the nonenveloped rhinovirus is one of the very resistant pathogens such as poliovirus and hepatitis A virus [37].

**A.**  **Interaction of Polyphenols of Seaweed with Enveloped Viruses**

**Figure 2:**
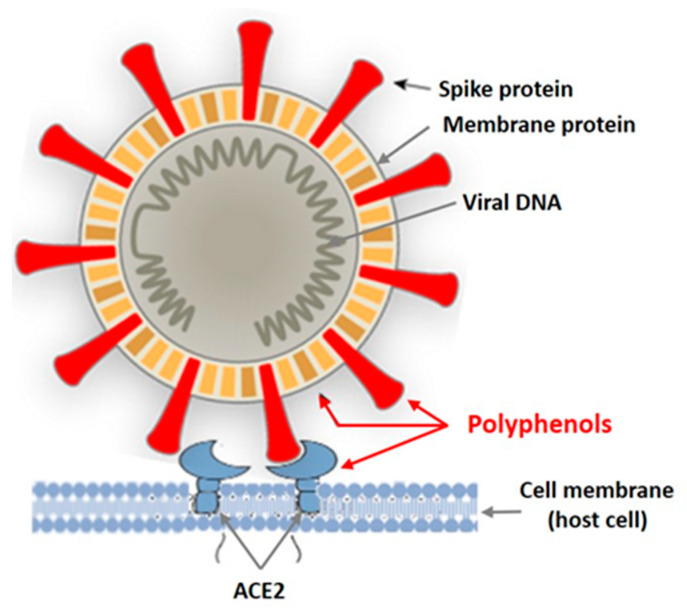
Targets of the enveloped virus for polyphenols of marine and terrestrial plants.

In recent years, intensive studies of the antiviral activity of polyphenolic compounds from terrestrial plants, as well as from various marine aquatic organisms, including macroalgae, have been carried out [38,39,40,41]. Mainly enveloped viruses are reported as sensitive to PPs. Figure 2 shows the targets of the enveloped virus that can be affected by plant polyphenols.

Tannins are known as powerful protein inactivators, including viral ones. M. Wink [38,39] showed that plant tannins form several hydrogen and ionic bonds when interacting with a virus protein, which act on the three-dimensional structure of the protein, suppressing its activity. As land plant tannins and algal tannins are similar in structure, the mechanisms of their interaction with enveloped viruses are probably similar. Polyphenols bind to viral envelope proteins, preventing the pathogen from interacting with the host cell.

Coronaviruses are enveloped viruses. To date, 39 known species of enveloped viruses are known, with each species comprising dozens and hundreds of strains. In addition to the nucleic acid and the associated structurally protective protein (in coronaviruses, it is the N protein), they also have a membrane envelope. The life cycle of coronaviruses provides many potential targets for antiviral intervention. Approaches to the development of anti-coronavirus drugs include exposure to the virus during the steps of penetration and entry of a viral particle into a cell, replication of viral nucleic acid, release of virion from a cell and effects on the cellular targets of the host.

One of the members of coronaviruses is the porcine epidemic diarrhoea virus (PEDL). First recorded in the United States in 2013, it has caused major economic damage in many countries due to the significant mortality of newborn piglets. The PEDL infects the cells lining the pig’s small intestine, causing severe epidemic diarrhoea and dehydration [40,41].

The causative agent was investigated using electron and immunoelectron microscopy. It was shown to differ from the coronaviruses known by that time: the porcine transmissible gastroenteritis (TGS) virus and porcine hemagglutinating encephalomyelitis. Kwon et al. [42] found an antiviral effect of ethanol extract and five phlorotannins obtained from the brown alga *Ecklonia cava* against the PEDL. The extracted compounds were identified as phloroglucinol (1), eckol (2), 7-phloreckol (3), phlorofucofuroeckol (4) and dieckol (5). Compounds (4) and (5) were present in the ethanol extract from seaweed in sufficiently large amounts [29].

To assess the antiviral activity of the compounds in vitro, two strategies were used: blocking the virus’ binding to cells (obtaining the effect of treatment simultaneously with the infection) and inhibiting the virus’ replication (obtaining the effect of treatment after the infection). The use of the former experimental scheme made it possible to establish that compounds (2–5) have an antiviral activity against the PEDL with the 50% inhibitory concentration (IC_50_) in the range from 10.8 ± 1.4 to 22.5 ± 2.2 μM. Compounds (2–5) completely blocked the binding of virus protein to sialic acid at concentrations lower than 36.6 μM by inhibiting hemagglutination. The results of the use of the latter experimental design showed that these compounds also blocked the virus’ replication with IC50 values of 12.2 ± 2.8 and 14.6 ± 1.3 μM, respectively, by inhibiting the synthesis of RNA and virus protein, but did not suppress the viral protease [28,30,31]. Regarding the cytotoxicity of the extract, the CC_50_ was 533.6 μg/mL and ranged from 374.4 to 579 μM for compounds (4) and (5). The experiments were carried out using the lowest toxic (>90% cell viability) concentrations of the extract [30,31].

The PT activity was distributed as follows: dieckol (16.6 ± 3.0 μM) > 7 phlorofucofuroeckol (18.6 ± 2.3 μM) > eckol (22.5 ± 2.3 μM). Phloroglucinol was inactive. PT activity was distributed as follows: dieckol (16.6 ± 3.0 μM) > 7 phlorofucofuroeckol (18.6 ± 2.3 μM) > eckol (22.5 ± 2.3 μM). Phloroglucinol was inactive. PT activity was influenced by the number of hydroxyl groups. Thus, oligomerisation and the existence of the cyclopentane ring may be important for the manifestation of antiviral activity. The authors recommend phlorofucofuroeckol and dieckol from the brown seaweed *E. cava* as potential agents that act on the most important targets of PEDV.

**B.**  **Interaction of PTs of Algae with Nonenveloped Viruses**

However, enveloped viruses are not only sensitive to the action of plant phenolic compounds, in particular tannins. Ueda et al. [43] found, for example, that persimmon extracts containing about 22% tannin reduced the infectivity of nonenveloped viruses (poliovirus, Coxsackie virus, adenovirus, rotavirus, feline calcivirus and mouse norovirus) by more than 4 log. The authors believe that the main mechanism of the antiviral action of the extract is associated with the aggregation of viral proteins, as evidenced by the competitive suppression of the antiviral effect by BSA. Algal phlorotannins also have an inhibitory effect on nonenveloped viruses. Such results are noted for human papillomavirus (HPV). As an example, we consider HPV, a small, nonenveloped virus possessing a capsid with cubic symmetry and containing two proteins, L1 and L2. The former is the main capsid protein that makes up more than 80% of the capsid material, forming blocks (capsomeres) from which the capsid is built. Anti-L1 antibodies exhibit virus-neutralising activity. L2 is a minor protein involved in the capsid stabilisation and linking with the genome [44]. The genital infection caused by the human papillomavirus (HPV) is the most common sexually transmitted disease. Most cases of cervical cancer are associated with this infection. Therefore, there is considerable interest in new effective non-reactogenic drugs for the treatment and prevention of this disease.

Kim and Kwak [44] investigated the effect of PT from the brown alga *E. bicyclis* on HPV. It was found that the seaweed EtOH extract exhibited antiviral activity against HPV 16PVs and HPV 18PVs. Then, the extract was sequentially separated with CH2Cl2, EtOAc and n-BuOH. The most active EtOAc fraction was used for chromatographic separation and resulted in the isolation of eckol, 8,8′-bieckolm 6,6′-bieckol and phlorofucofuroeckol A- Antiviral activity was assessed in 293T cell culture using bioluminescence. All compounds showed a decrease in the viral load of both viruses at a concentration of 50 μg/mL.

Noroviruses, a nonenveloped type of enterovirus, are considered the leading cause of epidemics of diseases accompanied by vomiting, diarrhoea, mild fever, abdominal cramps and nausea [45,46]. Norovirus is characterised by a long isolation period, low infectious dose, high resistance, considerable diversity and frequent genome mutations. The virus is transmitted through contaminated water or food and is spread by the faecal–oral route following contact with infected materials. The virus has a single-stranded positive sense RNA genome [47]. In recent years, attempts have been made to find harmless means of therapy and prevention of infection among terrestrial and marine organisms and algae [48]. To this aim, Eom et al. [40] investigated the possibility of using *E. bicyclis* seaweed extract and its ingredients as an alternative agent against norovirus. The following fractions were obtained from the EtOAc-soluble extract of *E. bicyclis*: phlorofucofuroeckol A (PFE) and dieckol (DE).

The MeOH extract and its components did not show significant cytotoxicity. The CC_50_ was 322.48 to 2146.42 μg/mL. The EtOAc extract showed strong antiviral activity and low cytotoxicity. Earlier [40], the authors described the structure of the extract components DE and PFE and their pronounced antiviral properties. PFE inhibits norovirus infection more intensely than DE. The selective index (SI) values for DE and PFE were approximately 20- and 25-fold higher than that of green tea epigallocatechin gallate. The antiviral activity of DE at IC_50_ was 0.9 ± 0.06, SI—CC_50_ IC_50_—550.6 ± 6.09; PFE, IC_50_—0.9 ± 0.07, SI—668.87 ± 73.06 [49].

The results obtained by the authors indicate that the use of PTs from *E. bicyclis* seaweed against norovirus infection is promising. They suggested that PTs prevent viruses from attaching to host cells and proposed to conduct an in-depth study of the mechanisms of anti-rotavirus action of these compounds.

An extract and PTs (eckol and PFE) from the seaweed *E. cava* were used to enhance protection against the nonenveloped RNA haemorrhagic septicaemia virus (VHSV) causing a highly contagious disease of freshwater and marine fish at different ages [41]. Using cell culture from fathead minnow, it was found that the extract and PTs at low concentrations exhibited strong antiviral activity. When cells were treated with the extract and PT simultaneously with the infection, the values increased (46.4–96.4%) as compared with those in the variants of the experiment before (16.5–48.4%) and after the infection (39.5–56, five%). The IC_50_ for the extract, eckol and PFE were 4.76 μM, 1.97 μM and 0.99 μM, respectively. The effect increased depending on the time of exposure. In in vivo experiments, a seaweed extract, administered orally at different doses to VHSV-infected flounder, increased the survival rate of fish (by 31.57% at a dose of 500 μg/g/day; by 12.5% at 50 μg/g/day) 12.5%) [40].

Thus, not only enveloped, but also nonenveloped viruses, are sensitive to seaweed PTs. The mechanism of action of these compounds towards the former is better known.

## 4. Seaweed Polyphenols and Their Inhibition of Vital Viral Proteins

An ideal antiviral agent should target the inhibition of key proteins involved in the pathogen’s life cycle. Potential inhibitors of these structures of viruses are polyphenolic compounds of seaweed, and, in particular, PTs.

Currently, the spread of the SARS-CoV-2 coronavirus is a serious public health problem, the solution of which requires the development of effective and harmless drugs. Coronavirus proteins are translated by one long polyprotein, from which two proteases are released: Mpro (major protease) and PLpro (papain-like protease). The active site of SARS-CoV 3CLpro contains a catalytic dyad consisting of Cys145 and His41, where a cysteine residue (Cys145) acts as a nucleophile and a histidine residue (His41) acts as a common acid base in the proteolytic process.

The central role of this protein in SARS-CoV replication has made it a major potential target for the development of antiviral drugs. Inhibition of this enzyme blocks SARS-CoV replication and enhances the antiviral response [50,51,52]. PLpro plays a role in the maturation and release of new viral particles from the cell, as well as in inhibiting the production of type 1 interferon synthesised by cells for protection. Suppression of interferon synthesis occurs by the action of PLpro on the ISG-15 gene in cellular proteins [53].

As the SARS-CoV proteases (3-chymotrypsin-like protease 3CLpro and papain-like protease PLpro) are synthesised as large precursor proteins that are cleaved to form mature active proteins, and their structures are retained in all genera of coronaviruses, the substances targeted at these proteins may be an effective strategy for the treatment of coronavirus infection by suppressing the viral genome replication [53,54].

In recent years, a number of phenolic compounds have been isolated from terrestrial plants, with inhibitory activity against the PLpro of coronaviruses, with inhibitory activity against S, the protein responsible for the fusion of the virus and the host cell prior to its penetration, as well as with inhibitory activity against replication pathogen [55,56,57]. However, even studies of polyphenolic compounds of terrestrial plants as anti-coronavirus agents, despite very encouraging results, are still at the experimental stage [58]. At the same time, the results of these studies allow researchers to hope that seaweed PPs may be more effective antiviral agents than polyphenols of terrestrial plants.

Park et al. [57], for the first time, studied PTs from the seaweed *E. cava* as an inhibitor of PLpro of the SARS-CoV virus. The authors obtained nine PTs from the ethanol extract from the seaweed. In the experiments using cell-free analysis, it was found that eight PTs (triphlorethol A, eckol, dioxinodehydroeckol, 2-phloreckol, 7-phloreckol, fucodiphlorethol, dieckol and phlorofucofuroeckol A) were dose-dependent competitive inhibitors of SARS-CoV 3CLpro. The IC_50_ values varied from 2.7 ± 0.6 (dieckol) to 164.7 ± 10.8 μM (triphlorethol A). The best inhibitory effect was exhibited by dieckol, which has two eckol groups linked via diphenyl ether [57].

The PTs of seaweed interact with vital proteins of other viruses, in particular with influenza virus neuraminidase (NA). Neuraminidase and hemagglutinin of the pathogen determine the antigenic properties of this pathogen. Hemagglutinin initiates infection by binding of the virus to α-2,6-sialic acid and/or α-2,3-linked sialic acid receptors on the host cell surface, followed by receptor-mediated endocytosis into the cell [59]. The sialic acid receptor on the surface of the host cell is a commonly recognised target for the development of broad-spectrum antiviral agents. Sialidase hydrolyses sialic acid on the cell surface and prevents the virus from attaching to cells. The NA protein serves as a sialidase and cleaves the bond between sialic acid and the HA protein to release virus particles. Neuraminidase thus plays a critical role in the life cycle of the influenza virus and also serves as an attractive target for the development of anti-influenza drugs [59]. PTs of seaweed can be used as candidates for the creation of such preparations.

Algae-derived PTs are considered candidates for such agents. In their work, Cho et al. [44] studied PTs from the seaweed *E. cava*, which is widely used as food in Asian countries, in particular Japan and Korea. Its main components are phlorotannins and fucoidan [60]. The authors investigated the antiviral activity against the influenza virus of 13 PTs obtained from 80% MeOH-extract of algae, which contained in their structures at least one fragment of 1,4-dibenzodioxin and were found mainly in Ecklonia and some other species seaweed [61,62]. Phlorofucofuroeckol A at IC_50_ = 13.48 ± 1.93 μM showed the highest antiviral activity against two strains of influenza A virus (H1N1 and H9N2). Six PTs showed a sufficiently high or moderate antiviral activity against both virus strains at a concentration of 20 μM. The compounds with high antiviral activity were tested for the synthesis of the viral protein of the H1N1A/PR/8/34 virus compared that of ribavirin as a positive control. Phlorofucofuroeckol A was tested on Madin–Darby canine kidney (MDCK) cells at concentrations of 5, 10, 20 and 40 μM. The compound more effectively inhibited viral protein expression in infected cells. In addition, this phlorotannin reduced the expression of NA and HA at a dose of 10 μM, and its strongest inhibitory activity was observed at a dose of 40 μM.

In the work of Ryu et al. [63], an ethanol extract from *E. cava* was fractionated, and five PTs were isolated. The extract showed a strong anti-neuraminidase activity (71.1% inhibition at a dose of 30 μg/mL). The inhibitory activity was studied on various strains of the influenza virus. Eckol showed a moderate IC_50_ value (89.5 μM) against the influenza A/Bervig-Mission/1/18 (H1N1) virus, but was inactive towards other viral strains (IC_50_ > 200 μM) compared to the other compounds tested (7-phloreсkol, phlorofucofuroeckol, and dieckol). The IC_50_ value of the compounds increased with an increase in the number of hydroxyl groups (from eckol to dieckol), which indicates the significance of this trait in NA inhibition. All the studied PTs were selective NA inhibitors [63].

Thus, phlorofucofuroeckol A from brown seaweed *E. cava* plays a key role in the antiviral activity of these algae against influenza viruses H1N1 and H9N2 and may be the basis for the further development of anti-influenza drugs, dietary supplements for food and functional food products.

Acquired immunodeficiency syndrome (AIDS) caused by the human immunodeficiency virus (HIV) is a major public health problem worldwide, especially in developing countries [64]. The human immunodeficiency virus (HIV) belongs to the family Retroviridae and the genus *Lentivirus*. It is an RNA enveloped virus with an unusual method of replication of genetic material. *Lentiviruses* (in Latin, “lente” means “slowly”) can cause diseases with a long incubation period and a slow, but steady progressive course. The cycle of their reproduction is characterised by a reverse flow of genetic information, i.e., DNA synthesis is carried out on a viral RNA matrix using an enzyme, reverse transcriptase (RT). RT, being vitally important for the virus, is a heterodimer (a protein of two polypeptide chains), consisting of two subunits (p66 and p51) [64].

Currently, the following antiviral drugs that act on various targets of the virus are available: nucleoside RT inhibitors, non-nucleoside RT inhibitors, protease inhibitors, integrase inhibitors, fusion inhibitors and antagonists of chemokine receptors (Table 1).

Unfortunately, HIV resistance to drugs is increasing daily; in addition, many of these agents have adverse side effects. For this reason, scientists’ attention has been attracted by new-generation drugs, to which HIV would not form resistance. Noteworthy and promising results were obtained in a study of anti-HIV properties of algae-derived PTs.

Ahn et al. [65] showed that 8,8-bieckol and 8,4 dieckol from the brown alga *E. cava* inhibit reverse transcriptase and HIV-1 protease, while eckol and phlorofucofuroeckol A from this alga did not exhibit such activity. 8,8-Bieckol and 8,4 dieckol more efficiently inhibited RT than protease. The IC_50_ of dieckol towards RT was 0.51 μM and was comparable to that of a reference drug, nevirapine (IC_50_ = 0.28 μM) [65]. 8,8-Bieckol and 8,4 dieckol more efficiently inhibited RT than protease. The IC_50_ of dieckol towards RT was 0.51 μM and was comparable to that of a reference drug, nevirapine (IC_50_ = 0.28 μM) [65]. Furthermore, the authors obtained diphlorethohydroxycarmalol, a carmalol derivative, from the marine brown alga *Ishige okamurae*. This compound had an inhibitory effect on RT and HIV-1 integrase with IC_50_ values of 9.1 μm and 25.2 μm, respectively. However, this compound did not have the same effect on HIV-1 protease. Acetylation neutralised this effect. 6,6′-Bieckol from *E. cava* reduced the cytopathic effects of HIV-1, including HIV-1-induced syncytium formation and p24 antigen levels.

Artan et al. [66] isolated 6,6′-bieckol, a phloroglucinol derivative, from the alga *E. cava* and characterised the compound by NMR. This phlorotannin showed a strong inhibition of HIV-1-induced syncytium formation (IC_50_ = 1.72 μM), viral p24 antigen production (IC_50_ = 1.26 μM) and lytic effects (IC_50_ = 1.23 μM). The compound selectively inhibited the activity of HIV-1 reverse transcriptase at IC_50_ = 1.07 μM, as well as the entry of HIV into cells. The authors proposed 6,6′-dieckol as a candidate for a new-generation drug against HIV infection [66].

Additionally, Karadeniz et al. [67] reported the anti-HIV activity of 8,4′-dieckol, a phloroglucinol derivative of *E. cava*. The compound dose-dependently inhibited the cytopathogenic effects of HIV-1, including HIV-1-induced syncytium formation in C8166 cells; suppressed lytic effects; and reduced the production of the viral p24 protein by H9 cells. Like the above-described agents, 8,4′-dieckol inhibited reverse transcriptase and viral penetration. However, it was found that, over time, the amount of syncytium in infected C8166 cells increased, the inhibitory activity was lost and phlorotannin had to be reintroduced into the cell culture [67].

It is important to emphasise that this compound suppressed replication of a virus with resistance to three drugs when cells were treated within 6 h post-infection. Similar results were obtained for nevirapine, the reverse transcriptase inhibitor. The authors suggest that the studied phlorotannin exhibits the effect of HIV-1 reverse transcriptase inhibition, possibly due to the binding of RT to sites or conformations other than those of virapine. This phlorotannin is a promising drug for the further development of new agents against HIV-1 with a pronounced efficacy compared to drugs available on the market [68]. Thus, using the example of three pathogens of most widespread viral infections, we have shown algal PTs to be promising for the creation of anti-HIV drugs whose targets are enzymes vital for viruses. In addition, these compounds also inhibit such viral functions as replication, entry in cell, syncytium formation, etc.

Note, however, that the degree of bioavailability of PT and individual differences in metabolism are significant limitations of their use. In addition, there are currently no analytical standards for the study of PT, and the exact relationship between the structure of compounds and their bioactivity is also unclear [67,68]. The authors suggest that the efficacy of medicinal plants against COVID-19 is not yet sufficiently demonstrated by studies, although some of them exhibiting IC_50_ below 10 μM can be considered promising, as they are capable of blocking viral proteins associated with its life cycle. After obtaining clinical evidence of the useful properties of algal PTs, these compounds in the form of natural products or biologically active substances can be combined with approved drugs against pathogenic viruses, which may be a promising alternative for the prevention and treatment of infections caused by them [68].

## 5. Synergism of Algae-Derived Phlorotannins and Antiviral Drugs

In viral infections, the simultaneous effect of drugs on several targets of the causative agent is of great importance, and therefore combined therapy has a number of advantages that allow:

- reduction in individual doses of drugs;
- reduction in the number and severity of side effects of antiviral drugs; and
- prevention, in some cases, of the emergence of drug-resistant virus variants [69].

The combination of targeted technologies with the inclusion of natural biologically active substances in the treatment regimen has shown numerous advantages of this therapeutic approach.

Although measles is a controllable infection, it takes thousands of children’s lives each year even in developed countries, and therefore the search for new natural compounds for the prevention and treatment of this infection continues. Moran-Santibanez et al. [70] investigated the effectiveness of the combined use of seaweed extracts rich in PPs and sulphated polysaccharides derived from the same seaweed and ribavirin against the measles virus. The authors used extracts from two seaweeds, *Ecklonia arborea* (class Phaeophyceae) and *Solieria filiformis* (phylum Rhodophyta), in experiments on a line of African green monkey kidney cells (Vero). Both extracts were characterised by low toxicity, high (compared with ribavirin) antiviral activity and high selectivity index (>3750 and >576.9, respectively). The selectivity index is the ratio of the 50% toxic concentration of a drug to its 50% virus-inhibiting concentration [70].

The extraction was carried out in accordance with the method described by Xi et al. [71] with six (*Ecklonia*) and five (*Solieria*) fractions obtained from the extracts. Phlorofucofuroeckol A was obtained from *E. arborea*. All samples used in the experiments were non-toxic to cells at the concentrations tested (from 0.1 to 1500 μg/mL). Ribavirin exhibited cytotoxicity starting with the 50% cytotoxic concentration (CC_50_) = 405 μg/mL.

All the tested components exhibited antiviral activity, which was assessed by determining the decrease in syncytium formation at various concentrations of the compounds (0.01, 0.1, 1 and 5 μg/mL of each extract and 10, 20, 30, 40 and 5 μg/mL ribavirin).

The combined effect was also assessed by determining the reduction in the syncytium formation. A combination of PT from *Ecklonia* and *S. filiformis* with sulphated polysaccharides (SPS) from *Solieria* showed the best synergistic effects, which was confirmed by PCR analysis. The best result was obtained by using PP from *Ecklonia* at IC_50_ and *Solieria* at IC_25_ + SPS from *Solieria*. All combinations with ribavirin were antagonistic. The authors noted that phlorotannins were most effective within the first 15 min post-infection, which suggests that this effect is due to direct inactivation of viral particles through preventing their adsorption and entry into cell.

The probability of viral entry into cells in the present of extracts was also determined. The best inhibitory effect was observed in the case of the *S. filiformis* extract compared to the control samples.

The virucidal activity of seaweed extracts is not only a preventive strategy to be implemented before a viral infection, but can also be an effective treatment after infection to prevent the virus from spreading over the body. Synergistic effect of ribavirin with PT with sulphated polysaccharides from the same seaweed can allow for a reduction in the concentration of drugs and thereby their cytotoxicity, as well as prevent the formation of resistance to therapeutic agents.

## 6. The Effect of PT on Pathogenetic Targets of Viral Infections in a Macroorganism

The role of the antioxidant properties of PT in the organism’s defence against viruses: Oxidative stress induced by a viral infection plays a significant role in the pathogenesis of infectious diseases [72]. Oxidative stress disrupts the balance between the production of free radicals, including reactive oxygen species (ROS), and the signalling pathways of antioxidant cells. It is a key factor in the pathogenesis of many acute and chronic viral diseases [73,74].

Reactive oxygen species (ROS) (such as superoxide radical anion (O-), hydroxyl radical (HO-) and nitric oxide (NO) and potential endogenous prooxidants, such as hydrogen peroxide (H_2_O_2_), hydrochloric acid (HC10), peroxynitrite (NО_3_^−^) and lipo-hydroperoxide (MOOH), have high reactivity, which damages the proteins, nucleic acids and lipids of the biological membranes of cells. Oxidative stress is a key factor in signal transmission by inflammatory cells for the regulation of cytokines and growth factors, as well as for immunomodulation and apoptosis [75]. It is known that oxidative processes contribute to viral replication in infected cells [76] and have an effect on the inhibition of cell proliferation and induction of apoptosis [77]. Thus, in patients infected with herpes simplex virus [78], the increased peroxidation of membrane phospholipids, induced by ROS, causes dysfunction of vital cell processes such as membrane transport and mitochondrial respiration [79]. The green tea component epigallocatechin has been shown to block the entry of HIV [80] due to its antioxidant properties.

Chen et al. [80] reported that infections caused by the Epstein–Barr virus (EBV) cause an increase in DNA damage and a significant accumulation of ROS; however, the use of free radical scavengers reduced the intensity of damage both in cells stimulated by the mitogen and in cells infected by this virus. A suggestion has been made that antioxidants counteract the damaging effects of reactive oxygen and nitrogen species, including free radicals, and therefore prevent or have a therapeutic effect on diseases associated with oxidative stress [81].

Modern medicine seeks to use various antioxidants to combat oxidative stress in viral infections. There is much evidence for the ability of natural antioxidants to trap ROS in infected cells, inhibit proapoptotic factors and thus restore intracellular balance between stress-related proteins (N-terminal kinases with Jun-JNK0 and promitotic (MAPK) and transcription factors NF-kB) [82,83,84]. Seaweeds are a rich source of antioxidants including PTs [76].

Viruses can be the causative agents of neuroinfections, directly mediating oxidative stress, the central link of which is the peroxidation of lipids, which play a key role in the nervous system [72]. Thus, the beneficial properties of phlorotanins are associated with their properties as a powerful antioxidant, anti-inflammatory and immunoregulatory molecule, as well as with their neuroprotective effect.

In neurotropic flavivirus infections (such as JEV, WNV and TBEV infections), oxidative stress is an important component of neuroinflammation [85,86]. When various neuronal cell lines were infected with flaviviruses, the increase in ROS production induced uncontrolled activation of microglia and neuronal death [87]. Being ROS scavengers, polyphenolic compounds from seaweeds are considered as potent antioxidants. In this regard, the polyphenolic complex luromarin derived from the seagrass *Zostera marina*, containing phenolpropanoid and flavone, is of great interest. Note that phenolpropanoid, in terms of antioxidant activity, is noticeably superior to all known antioxidants [85,86,87].

Studies [88] have provided data on in vitro and in vivo studies of the antiviral efficacy of a PP complex isolated from seagrasses of the family Zosteraceae, which are flowering plants adapted to living in saline water of seas and oceans.

The antiviral activity of luromarin and its components against a highly virulent strain of tick-borne encephalitis (TBE) virus was studied in vitro. It was found that the exposure to these compounds at 1 h before the infection of the cells had no effect on the reproduction of the virus. A different result was obtained by the authors in the study of direct virucidal action (preliminary incubation of the compounds under study with the virus for 1 h before cell infection) [89,90,91]. Thus, the main mechanism of action of the natural antioxidants (that make up luromarin) and the entire complex against the TBE virus is the direct inactivation of viral particles and inhibition of the TBE virus at an early stage of replication.

In a model of acute TBE infection in mice, oral administration of luromarin and its components at 1 h after subcutaneous infection of animals provided 30–35% protection by increasing their lifespan by 2–3 days compared to the control. The authors explain this by the complex protective effect of luromarin and its components, which have not only a selective effect on various phases of viral infection, but also a systemic effect on the body due to their high antioxidant, anti-inflammatory and neuroprotective potential.

### The Role of Anti-Inflammatory Action of Polyphenols in Protection against Viral Infections

Inflammation is a complex process regulated by a cascade of various proinflammatory cytokines, growth factors, nitric oxide and prostaglandins produced by activated macrophages [92]. With inflammation, the affected tissues become infiltrated by macrophages, and the disposal of decay products, repair and regeneration occur. The anti-inflammatory effect of phenolic compounds is directly related to their antioxidant activity against ROS [93].

The cytokine production and secretion are among the earliest events accompanying the interaction of microorganisms with macrophages. This early non-specific response to infection is important for the organism. It develops very quickly, as it does need a clone of cells that respond to a specific antigen. The early cytokine response influences the subsequent specific immune response.

The contact of a virus with an organism is accompanied by the production of interferon, a soluble factor produced by virus-infected cells. Interferon is capable of inducing antiviral status in uninfected cells and makes them unsuitable for viral reproduction. Interferon activates macrophages which begin to synthesise IFN-γ, IL-1, IL-2, IL-4, IL-6 and TNF-α; as a result, macrophages acquire the ability to lyse cells infected by the virus. At the same time, interferon is able to induce expression of more than 100 different genes in the macrophage genome [94].

In some cases, e.g., in severe cases of coronavirus infection (COVID-19), a “cytokine storm” develops. It is an inflammatory response of organism, with the level of cytokines in blood increasing sharply, which causes the immunity to attack cells and tissues of own organism. A consequence of this response can be the destruction of tissues and organs and, as a result, the death of the organism.

Seaweed PPs are not only antiviral, but also potent anti-inflammatory compounds. For example, 8,8′-dieckol from the seaweed *E. cava* inhibited the production of nitric oxide, a key mediator of inflammation, and prostaglandin E2 (PGE2) by macrophages stimulated by lipopolysaccharide. This compound inhibited the production of nitric oxide by suppressing the expression of inducible nitric oxide synthesis (iNOS). Phlorotannin reduced the production and expression of IL-6 mRNA, but did not inhibit TNFα. The exposure of macrophages to this PT decreased the NF-κB transactivation and the nuclear translocation of the p65 NF-κB subunit and suppressed the lipopolysaccharides (LPS)-induced production of intracellular ROS in macrophages. Thus, the anti-inflammatory properties of PT are associated with the suppression of NO, PGE2 and IL-6 through the negative regulation of the NF-κB pathway and ROS production in RAW264 macrophages [95].

The range of anti-inflammatory effects may vary between different PTs. Thus, phlorofucofuroeckol A from the alga *Eisenia bicyclis* also exhibited an anti-inflammatory effect in the same model. In addition to reducing NO and PGE2, phlorotannin dose-dependently inhibited the production of COX-2 cyclooxygenase by macrophages, and also reduced the production of the proinflammatory chemokine MCP-1 [84]. These authors, as well as previous ones who worked with other PTs, showed that phlorofucofuroeckol A exerts an anti-inflammatory effect by blocking the NF-κB and MAPK signalling pathways in RAW264.7 macrophages stimulated by LPS. Eckol, a compound widely distributed in the brown seaweed *Ecklonia*, has attracted scientists’ interest as an anti-inflammatory agent [96,97,98].

The inflammatory process is an inevitable consequence of viral infections. In this regard, algal PPs, which have both antiviral and anti-inflammatory effects, are promising objects for the development of drugs, biologically active food additives and functional food products on their basis.

## 7. In Vivo Efficacy of Polyphenolic Compounds

The results obtained under in vitro conditions are not always adequate to those obtained under the conditions of a macroorganism. For polyphenols, in many cases there is no direct correlation between the results of in vitro experiments and clinical trials, and the interpretation of experimental data should be treated with great caution.

Almost all in vitro experiments use flavonoid aglycones or polyphenol-rich extracts. In this regard, it can be assumed that under the conditions of a macroorganism, target organs almost never come into direct contact with aglycones of flavonoids, but only with their metabolites or conjugated forms [99,100]. In addition, the concentrations of aglycones that are commonly used in vitro experiments are almost never achieved in vivo. Therefore, after consumption of a single polyphenolic compound in doses of 10–100 mg, its maximum concentration in blood serum, as a rule, does not exceed 1 mM. Moreover, with rare exceptions, native flavonoids (aglycones) in the blood usually cannot be determined [99,100,101,102].

This section presents the results of an in vivo study of the antiviral activity of several polyphenolic compounds, which, according to other indications, are already used in medicine with positive effects in the form of medicines or biologically active food additives. They are presented by us as proof of the prospects of this group of biologically active substances for use as antiviral agents of a new generation with different mechanisms of action than synthetic drugs.

Quercetin and its derivatives: Quercetin, like other flavonoids, is a polyphenolic compound, the main structural element of which is composed of two aromatic rings, A and B, connected by a three-carbon bridge, forming a pyran or pyrone (in the presence of double bonds) ring [103]. This group of compounds has long attracted attention as potential therapeutic agents for the fight against respiratory tract infections [104].

Thus, Choi et al. [105] investigated the activity of quercetin-3 rhamnoside (Q3R) against the influenza A/WS/33 virus in mice. The animals received the drug orally (6.25 mg/kg per dose) two hours before infection and once a day for 6 days after infection with the influenza virus. In animals treated with Q3R, there was a significant reduction in weight loss and mortality. The titres of the virus in the lungs of mice in this group on day 6 after infection were about 2000 times lower than in animals treated with the control drug: oseltamivir. The use of the test drug delayed the development and progression of lung lesions. The authors believe that this compound may be a promising candidate for the development of anti-influenza drugs. As for the bioavailability of the compound, it is known that such derivatives are absorbed more efficiently than aglycone. A characteristic feature of the bioavailability of flavonols is their very slow elimination from the body (the half-life is from 11 to 28 h), which can contribute to the accumulation of metabolites in the blood plasma during repeated administration [106].

It was found that quercetin inhibits oxidative stress induced by the influenza virus [107]. The administration of quercetin to mice infected with the influenza A/Hong Kong/8/68 virus significantly reduced the level of lipid peroxidation in the animal body. In the lungs of such mice, the level of antioxidant enzymes—superoxide dismutase, catalase and reduced glutathione—increased [108]. Quercetin and rutin are recommended for inclusion in post-infection treatment [107,108].

To reduce the body’s susceptibility to upper respiratory tract infections after stressful physical exertion, a short-term inclusion of quercetin in the diet is recommended, which has been proven by convincing results from in vivo studies [109].

The efficacy of quercetin has also been demonstrated in vivo experiments for respiratory tract infections caused by rhinovirus, which causes most colds and is a common cause of exacerbations in patients with asthma and chronic obstructive pulmonary disease [110,111]. The authors injected mice intranasally with the rhinovirus RV1B, which causes inflammation and interferon (IFN) production [112]. After 2 h, the animals were injected with quercetin or propylene glycol (vehicle). After a day and 4 days, the mice were sacrificed and the viral load in the lungs was determined, respectively. The viral load in animals infected with rhinovirus was 9 × 10^4^ CC ID_50_/mL of virus. No virus was detected after 4 days [112]. The mice treated with quercetin had a viral load that was 4 log less in the RNA of the virus compared to animals that received only the virus. In this case, the replication of the pathogen occurs only on the first day after infection and quercetin effectively inhibits this process. In addition, it helps to reduce the inflammatory process—it lowers the levels of CXCL-1 (KC), CXCL-2 (MIP-2), as well as TNFα and CCL2 (MCP-1). In mice not treated with quercetin, elevated levels of all four cytokines/chemokines were observed. The therapeutic effect was more pronounced when quercetin was used simultaneously or after infection.

Thus, quercetin suppresses rhinovirus-mediated viral infection at several stages of the viral life cycle, including endocytosis, viral genome transcription and viral protein synthesis; thus, it may be useful in limiting pathogen replication and reducing disease symptoms. Moreover, it is a powerful antioxidant and has powerful anti-inflammatory properties.

Enteroviruses pose a serious threat to human health. They cause a variety of pathological processes from mild disorders to death. The number of safe and effective drugs against enteroviruses is small, and therefore there is a need to develop new anti-enterovirus drugs. Galochkina et al. [113] investigated the effect of dihydroquercetin (DHQ) on the course of pancreatitis in white mice caused by Coxsackie B4 virus (CVB4). DHQ is a natural biologically active substance obtained from the bark of Siberian larch. It is a bioflavonoid with powerful antioxidant and anti-inflammatory properties. DHQ is on the drug registry and is non-toxic even at very high doses. The drug was administered to mice intraperitoneally at doses of 75 or 150 mg/kg/day once a day for 5 days after intraperitoneal infection. Ribavirin was used for comparison. The use of DHQ led to a dose-dependent decrease in the titre of the virus in the tissue of the pancreas. The morphology of the gland tissue of the animals receiving DHQ was less pronounced than in the control animals, displaying infiltration with inflammatory cells and no signs of destruction. The glandular tissue of mice treated with both DHQ and ribavirin had fewer inflammatory foci, and the latter, in turn, contained fewer infiltrating cells than animals treated with placebo. The effect was comparable to or superior to that of ribavirin. The authors concluded that DHQ is promising for use in the complex treatment of viral pancreatitis [113].

Another quercetin derivative, quercetin 3-β-O-D-glucoside, has been shown to be effective against the Ebola virus. This compound protected ABD2F1/Jena mice from intraperitoneal infection with Col.Sk, MM, MengoM, L viruses, but did not protect against intracerebral infection. The drug protected mice from Ebola even when administered just 30 min before infection, which allowed the authors to position the compound as having potential as a prophylactic agent against Ebola virus infection [114]. However, more serious research is required to finally determine the effectiveness of the compound for different modes of administration, different doses, etc.

Thus, a significant number of studies in vivo carried out both in animals and with the participation of patients show that quercetin is a promising candidate for combination therapy for various viral infections [115]. The above materials make it possible to consider the use of quercetin and its derivatives as promising for viral infections of various aetiologies. However, caution should be noted in extrapolating data from animals to humans [106].

Baikalin. The flavonoid baikalin is obtained from the roots of the *Scutellaria baicalensis*. In alternative medicine, it is used as a biologically active food supplement and in Asian countries as a drug. Currently, there is an extensive list of agents approved for use on the basis of or in combination with baicalin, the effectiveness of which has been proven for various indications in vivo. Many works carried out in vivo are devoted to the study of the effectiveness of baicalin in infections caused by different strains of the influenza virus [116,117,118].

Chu et al. [118] investigated in an experiment on mice the effect of baicalin on the human influenza A/PR/8/34 strain adapted to these animals. C57Bl/6 mice were inoculated with 0.1 LD_50_ (5 × 10^3^ PFU/mL) of influenza virus. All infected mice died by day 8. Mice that received different doses of baicalin (1.0 g/kg, 1.5 g/kg and 2.0 g/kg) survived by day 8 (70%, 80% and 80%, respectively), and 60%, 70% and 80% survived until day 14, respectively. They did not lose weight. Virus titres in mice untreated with baicalin on day 7 increased to 106.3 pfu/mL. The titres of the virus in those who received the test preparation were, respectively, 102.7, 102.3 and 102.2 pfu/mL. Hemagglutination titres in untreated mice were 1:640, and in groups receiving the compound were 1:80, which indicates the inhibitory activity of baicalin on viral replication. Baikalin also inhibited the development of the inflammatory process in the lungs. In the same experiments, it was found that baicalin induces the secretion of IFNγ, which determines the antiviral activity of this compound. The last statement is proved by the fact that this compound did not have an antiviral effect in animals with IFNγ gene knockout [118].

In another study [119], it was found that oral administration of baicalin to mice infected with the Sendai virus leads to a significant reduction in virus titres in the lungs of animals and protection from death.

Resveratrol. Resveratrol is a highly active polyphenolic compound that is currently being actively studied both in vitro and in vivo as an antiviral agent [120,121]. The antiviral effects of this compound are associated with inhibition of viral replication, protein synthesis, gene expression and nucleic acid synthesis. The antioxidant effect of resveratrol is manifested by inhibiting important gene pathways such as NF-kB.

Resveratrol is poorly soluble in water and has a low oral bioavailability, and therefore research is currently focused on the development of structured nanoparticles that can improve the bioavailability of this biologically active substance and prolong its in vivo release.

Resveratrol has been used to prevent airway inflammation and reduce airway hypersensitivity caused by respiratory syncytial virus (RSV) infection. Healthy mice are immune to this pathogen. However, immunocompromised animals (treatment with cyclophosphamide) become susceptible to the virus [120]. It was found that resveratrol suppresses viral replication in the lungs of these mice and the number of infiltrating lymphocytes present in the lavage bronchoalveolar fluid reduces inflammation. In addition, resveratrol significantly reduced lavage fluid IFNγ levels associated with RSV-mediated airway inflammation [120].

In addition, resveratrol was highly active against rotavirus, which is the main causative agent of viral gastroenteritis in infants and young children [121]. Using a model of suckling mice infected with rotavirus, the authors found that resveratrol supplementation significantly reduced the severity of diarrhea, reduced viral titres and improved clinical symptoms. In the tissues of mice treated with resveratrol, the levels of expression of mRNA, IL-2, IL-10, TNFα, IFNγ, MIP-1 and MCP-1 were significantly reduced. Experiments have shown the promise of resveratrol as a potential treatment for rotavirus infection [121].

We have shown only some of the results of an in vivo study of the effectiveness of certain plant polyphenols known to all, on the basis of which approved drugs and biologically active food additives were obtained. Analysis of modern literature shows that these compounds are being actively studied now in vitro, ex vivo and in animal experiments. There are still very few clinical evidence-based studies on the effectiveness of this group of herbal biologically active substances. In this regard, the results obtained by Matsumoto et al. [122]. The authors conducted a double-blind, placebo-controlled study with 200 health workers conducted over 5 months at three health facilities for the elderly in Higashimuraami (Japan). One group of patients (98 people) received green tea catechins (378 mg/day) and theanine (210 mg/day). The control group (99 people) received a placebo. Four participants in the catechins/theanine group and 13 in the control group contracted the flu. Thus, the consumption of green tea catechins and theanine had a distinct preventive effect on the incidence of influenza.

Currently, polyphenolic compounds from land and sea plants are being actively studied due to their high biological activity. However, scientists must look for ways to increase the bioavailability of these compounds. For this purpose, many studies have proposed structural derivatives of the starting compounds. Some studies suggest using nanoparticles to encapsulate polyphenolic compounds for greater efficacy. In addition, the administration of polyphenolic compounds with other antiviral drugs can improve their bioavailability. An example is the studies of O’Shea et al. [123]. The authors examined the efficacy of monoclonal antibodies (mAb; AR4A) and epigallocatechin gallate (EGCG) in vitro and in vivo. The combination therapy completely protected animals from HCV 1a genotype infection.

Regarding new polyphenolic compounds, which are currently being studied in large numbers all over the world, serious studies of their specificity, activity, bioavailability and safety are required before they are proposed as drugs. After careful testing and approval for use in humans, it can be recommended to use such compounds in the form of dietary supplements and nutritional supplements.

It is necessary to develop strategies for increasing the bioavailability of polyphenols, to determine whether these methods lead to an increase in biological activity in the body. The benefits and efficacy of polyphenols in viral infections should be demonstrated in appropriate animal and human disease models.

## 8. Conclusions

PPs are unique compounds found in seaweed at high concentrations. For example, *Ascophyllum nodosum* contains 14% PPs, compared to 2–3% in terrestrial plants. The high concentration of PPs in seaweed, combined with the simplicity of their cultivation, harvesting and processing, makes them attractive as a cheap source of pharmaceutical substances and a basis for creating dietary supplements for food and functional foods.

Algae-derived PPs (phlorotannins) combine several types of activities, each of which contributes to decrease in viral load, decrease in the intensity of the inflammatory process, increase in the antioxidant properties of blood and correction of immune disorders. It has been shown that these compounds affect different stages of the life cycle of viruses: they block the first stage (attachment of the pathogen to the cell surface) of viral infection, prevent the spread of the virus and its ability to develop and acquire drug resistance, and also in some cases have a direct antiviral effect. PTs inhibit viral replication by blocking vital viral enzymes and preventing the release of viral particles from the cell. Other types of biological activities, i.e., anti-inflammatory, antioxidant, immunomodulatory and antitoxic, have become vitally important in case of late detection of the disease, its severe or complicated form, where the leading role in pathogenesis is played by reactive processes rather than by viral ones, complicated by bacterial infection. It should be noted that algal PPs affect vital processes that are common to severe viral inflammatory processes, irrespective the aetiology of the disease: the production of proinflammatory cytokines, cell migration to the inflammatory focus, etc. Therefore, algal PTs can be a basis for the development of drugs effectively acting on the innate immunity in various viral infections.

A combination of target technologies has shown numerous benefits in combating viral diseases. PTs from seaweed can be used in combination with officially approved drugs, which makes it possible to reduce the dose of these therapeutic agents and thereby reduce side effects. However, algae-derived PTs have been insufficiently studied to date, mainly in experiments. In this regard, it is necessary to extend research on the bioavailability of these compounds obtained from various seaweed species and the spectrum of their antiviral activities [99]. It should be determined which seaweed PPs have the strongest antiviral effect and which work best: whole seaweed, extracts enriched in PPs or PTs with a fixed structure. Another important question is do algae-derived PPs alter the composition and functioning of the intestinal microbiota? Furthermore, at last, can there be any undesirable effects caused by long-term use of different algal polyphenols at high doses?

Currently, the presence of complex polymer mixtures of their structural and conformational isomers in seaweed poses a serious challenge to researchers to characterise the composition of PTs, which is absolutely necessary for the development of pharmaceutical products. For this reason, only a selective structural characterisation of PTs is still possible [13]. It is also necessary to understand to what extent the results obtained in experiments with PP from seaweed in vitro can be extrapolated to their actual effects in a whole organism, and whether the very effective, but still poorly studied, compounds can be considered an alternative or supplement to the existing strategies for the treatment of viral diseases. Relevant material is still being accumulated; the analysis of this material may subsequently create conditions for the creation of medicinal antiviral drugs with a new mechanism of action, resistance to which in pathogens would not form.

In the current conditions of the ongoing pandemic, the results of studies on the targeted effects of algal polyphenols on coronaviruses seem very promising, although these studies have been carried out in vitro so far. This issue becomes especially relevant due to the lack of effective drugs, including synthetic ones, for the treatment of viral diseases such as the coronavirus infection. It is important that polyphenols (as has been established for polyphenols from terrestrial plants) do not cause side effects and are also not antagonistic to drugs used for viral infections [100].

Effective nutraceuticals, to be potentially developed on the basis of algal polyphenols, can also be used in the complex therapy of viral diseases. It is necessary to extend in vivo studies on laboratory animals, which subsequently will allow proceeding to clinical tests.

Thus, studies in recent years have shown that algal polyphenols are polyfunctional compounds and, therefore, they have a great potential as active ingredients for the creation of novel pharmaceutical substances with antiviral activity.

## Figures and Tables

**Table 1:** Antiretroviral drugs that act on various targets of the virus.

Antiviral Drug Class	Antiviral Mechanism of Action	Examples of Available Drugs
Nucleoside/nucleotide reverse transcriptase inhibitors (NRTIs)	Affect the ability of a virus to multiply or reproduce. NRTIs prevent the virus’s reverse transcriptase from accurately copying its RNA into DNA.	Zidovudine (Retrovir), Lamivudine (Epivir), Abacavir sulfate (Ziagen), Didanosine (Videx), Stavudine (Zerit), Emtricitabine (Emtriva)
Non-nucleoside reverse transcriptase inhibitors (NNRTIs)	NNRTIs block DNA elongation by directly binding to the reverse transcriptase enzyme	Delavirdine, Efavirenz, Etravirine, Nevirapine, Rilpivirine
Protease inhibitors	Protease inhibitor drugs block the action of protease enzymes. This can stop the virus from multiplying.	Atazanavir (Reyataz), Darunavir (Prezista), Fosamprenavir (Lexiva), Indinavir (Crixivan), Nelfinavir (Viracept), Ritonavir (Norvir), Saquinavir (Invirase)
Integrase inhibitors	These drugs stop HIV from being able to make integrase, which is necessary for its replication.	Raltegravir (Isentress), Dolutegravir (Tivicay), Elvitegravir, Bictegravir
Inhibitors of fusion	Inhibitors of the fusion of HIV to host cells, preventing viral entry.	Enfuvirtide, Maraviroc, Leronlimab, Aplaviroc, Ibalizumab, Temsavir
Inhibitors of chemokine receptors	These drugs inhibit chemokine receptors (CXCR4 and CCR5) and block the entry virus into the host cell.	Selzentry (Pro) Maraviroc, Bicyclam derivatives, AMD070
